# Pore formation in p-type silicon in solutions containing different types of alcohol

**DOI:** 10.1186/1556-276X-7-329

**Published:** 2012-06-21

**Authors:** Tomoko Urata, Kazuhiro Fukami, Tetsuo Sakka, Yukio H Ogata

**Affiliations:** 1 Institute of Advanced Energy, Kyoto University, Uji, Kyoto, 611–0011, Japan

## Abstract

Macroporous structure of silicon can be obtained with anodization in hydrogen fluoride (HF) solution. The macropore formation in the presence of alcohol was studied. Macroporous layer formation in a low-concentration HF solution is stabilized with the increasing number of carbon in alcohol. The dissolution at the topmost part of the porous layer is observed though the behavior depends upon the type of alcohol. Meanwhile, the total mass loss of dissolved silicon is almost constant. Such dissolution at the top surface occurs only when the concentration of HF is low. Adding organic solvents to the HF solution also leads to the suppression of the pore wall dissolution. The type of alcohol and HF concentration in solution affect the formation of porous silicon.

## Background

Anodization of silicon in hydrogen fluoride (HF) solution below the critical current density produces a porous silicon layer. The size and shape of the pores are dependent on wafer resistivity, current density, and solution composition. According to the pore diameter, pores are mainly classified into three groups, micropores (<2 nm), mesopores (2–50 nm), or macropores (>50 nm). The macroporous structure in n-type silicon substrate was, for the first time, discovered using aqueous HF solution by Theunissen in 1972 [1]. However, it was in 1994 that Propst and Kohl observed first macropores in p-type silicon [2]. They used anhydrous HF solution with acetonitrile (MeCN) as electrolyte for this macropore formation. Later, further studies regarding macropores in p-type silicon were performed either in aqueous HF [3] or HF mixed with organic solvents [4].

HF solutions mixed with various organic solvents have become a widely used solution for the pore formation. For example, some organic solvents such as MeCN or dimethylformamide (DMF) facilitate macropore formation in p-type silicon. The effect of organic solvents has been discussed from various points of view over the years. Though Lehmann and Rönnebeck reported that the presence of an organic solvent in the electrolyte is not essential for macropore formation [5], it is generally accepted that organic solvents essentially suppress the electrochemical oxidation of silicon in comparison to an aqueous electrolyte [6]. The formation of macropore with MeCN is different from that with DMF and dimethyl sulfoxide [7]. Actually, it has been shown that some organic solvents can act as a mild oxidizing reactant for silicon [8].

For micro or mesoporous silicon formation, an electrolyte usually contains alcohol as a solvent. The effect of the presence of alcohol has been considered that it helps to reduce the surface tension or to keep away from coverage of H_2_ bubbles on the porous layer, but alcohol does not react with silicon directly. Alcohol also became to be used for macropore formation. But the effects of alcohol upon the pore formation have attracted little attention. In the present work, the effects of the presence of alcohol on porous layer formation in p-type silicon are studied.

## Methods

Single crystalline silicon wafers oriented to (100), used in this study, were p-type boron-doped with a resistivity of 10–20 Ωcm, and they were prepatterned. Etch pits orthogonally aligned at an 8 μm interval were created on the silicon wafer by standard photolithographic patterning and subject to subsequent alkaline etching in a 25 wt.% tetramethylammonium hydroxide aqueous solution for 5 min at 358 K. All wafers were rinsed in acetone and ultra pure water, and afterward dipped in 5 wt.% HF aqueous solution to remove the native oxide layer.

Electrochemical anodization was performed galvanostatically in a cell made of trifluoroethylene resin using a two-electrode setup with a platinum rod serving as the counter electrode. The ohmic contact was achieved by painting Ga-In alloy on the backside of the wafer with a copper current collector. The exposed surface area of the silicon electrode was 0.2 cm^2^. Anodization was carried out at 14 mA cm^−2^. Duration of the anodization was 1 h in all cases. The solution was a mixture of aqueous HF solution (47 wt.%), ultra pure water, and the following alcohols with a composition of 5:6:29 (ca. 8 wt.% HF) or 22:6:12 (ca. 30 wt.% HF) in volume: methanol (MeOH), ethanol (EtOH), 2-propanol (PrOH), and *t*-butanol (BuOH). In some experiments diethyl ether (Et_2_O), which has a much low polarity, was added. This solution contained 47 wt.% HF aqueous solution, ultra pure water, alcohol, and Et_2_O with a composition of 5:6:15:14 in volume, meaning that the concentration of HF was kept at a constant value.

The porous structures were observed using a field emission type scanning electron microscope (SEM, JSM-6500 F, JEOL Ltd., Tokyo, Japan). The profile of the surface was determined by a line scan using a pin-type profilometer (P-12, KLA-Tencor Corporation, Milpitas, CA, USA). The scan speed of 200 μm s^−1^, scan length of 12 mm, and stylus force of 10 mg were the main parameters for the scanning. SEM observations were also performed for the determination of the surface topography.

Mass loss of silicon dissolved during anodization (*Δm*_HF_) was measured with an ultra-microbalance (XP2UV, METTLER TOLEDO Co., Columbus, OH, USA). After dipping in 5 wt.% HF aqueous solution, the mass of silicon wafer was measured first (*m*_Sibefore_). After anodization, Ga-In alloy on the backside of the wafer was removed with HCl, then the mass of sample was measured again (*m*_Siafter_) after drying with Ar gas. *Δm*_HF_ was calculated as follows:

(1) $$\Delta m_{\text{HF}}=m_{\text{Sibefore}}-m_{\text{Siafter}}.$$

The apparent electrochemical valence, *n*, can be evaluated according to the following equation:

(2) $$\Delta m_{\text{HF}}=\left(M_{\text{Si}}\cdot Q\right)/\left(n\cdot F\right)$$

with *M*_Si_ being the atomic mass of silicon, *Q* the electrochemical charge, and *F* the Faraday constant.

## Results and discussion

**Figure 1:**
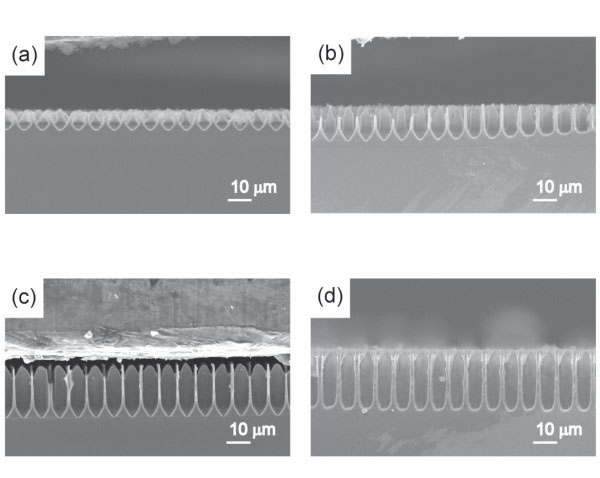
**SEM micrographs of macropores formed in HF solutions containing various types of alcohol.** Cross-sectional views of silicon anodized in a 10–20 Ωcm prepatterned p-type silicon at 14 mAcm^−2^ for 1 h in HF solutions containing (**a**) MeOH, (**b**) EtOH, (**c**) PrOH, and (**d**) BuOH.

Figure 1 shows cross-sectional views of porous layers anodized in ca. 8 wt.% HF solution with MeOH, EtOH, PrOH, or BuOH. When MeOH is used as solvent, pores are not obtained. With EtOH, pore walls start to be formed but the depth of pores are not deep. As discussed later, this is not due to the slow dissolution of silicon. When using PrOH or BuOH, pores are obtained comparatively well. The stability of porous layers increases with the increasing carbon numbers in the alcohol.

**Figure 2:**
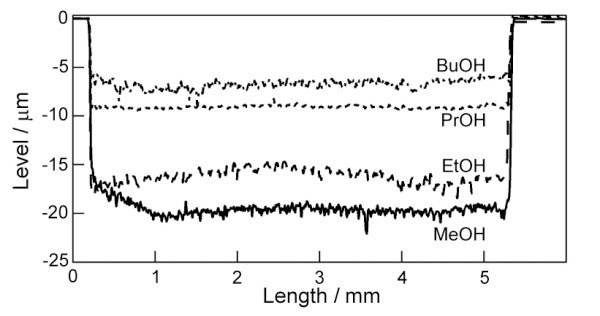
**Topographic profiles.** Cross-sectional profiles of silicon anodized in low-concentration HF solutions with MeOH, EtOH, PrOH, and BuOH.

When an edge of porous layer was observed with SEM, there was a difference of surface levels between the original wafer surface and the top surface of anodized part. The dissolution behavior of the top surface varies depending on the type of alcohol. The depth profiles of anodized surfaces are shown in Figure 2. The “0” in the ordinate indicates the original wafer surface determined as the level of the silicon surface which was not contacted with electrolyte and not anodized. The level of the top surface anodized in the MeOH solution is the deepest, while it is the lowest for the BuOH solution. The dissolution of the top surface is suppressed more with the increasing number of carbon atoms in alcohol.

The mass loss of silicon was measured after anodization. Table 1 shows mass loss of silicon after anodization in various types of alcohol. There is little difference among the types of alcohol contrary to our expectation. The apparent electrochemical valence was also calculated from the values of the mass losses.

**Table 1:** Mass loss of silicon during anodization

**n/C**_**n**_**H**_**2n+1**_**OH**	**1**	**2**	**3**	**4**
Mass loss of silicon/mg	1.13	1.14	1.16	1.18
Apparent valence	2.6	2.6	2.5	2.5

**Figure 3:**
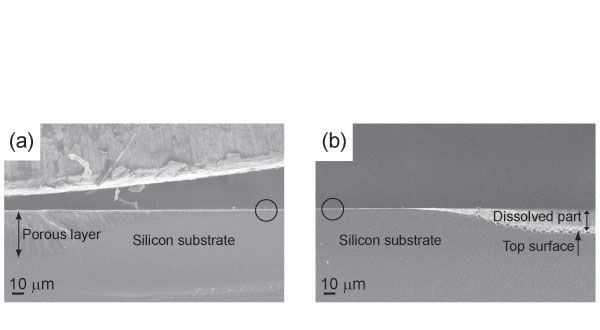
**SEM micrographs of macropores prepared in the MeOH solutions with different HF concentrations.** Cross-sectional views of porous layers anodized in 10–20 Ωcm prepatterned p-type silicon at 14 mAcm^−2^ for 1 h in HF: H_2_O:MeOH (**a**) 22:6:12 (ca. 30 wt.% HF) and (**b**) 5:6:29 (ca. 8 wt.% HF) in volume. Circles show the positions that have not undergone anodization.

In order to clarify the mechanism leading to the dissolution of the top surface, silicon was anodized in ca. 30 wt.% HF solution with alcohol at 14 mA cm^−2^ which was a current density for macropore formation. In our previous work, the top surface of the porous layer keeps the original level of the wafer when the solution contains 28 wt.% or higher HF with EtOH [9]. Using ca. 30 wt.% HF solutions with all kinds of alcohol we treated, the top surface of the sample did not dissolve appreciably. SEM images of the samples anodized in both ca. 30 wt.% and ca. 8 wt.% HF solutions with MeOH are shown in Figure 3. Circles in Figure 3 represent the original levels of the wafer which are not exposed to the solution. Using ca. 8 wt.% HF solution with MeOH, only a thin porous layer remains with considerably dissolved top part, while a thick microporous layer is obtained with ca. 30 wt.% HF solution. The dissolution is suppressed when the high concentration of HF solution is used even in the case of using MeOH solution.

**Figure 4:**
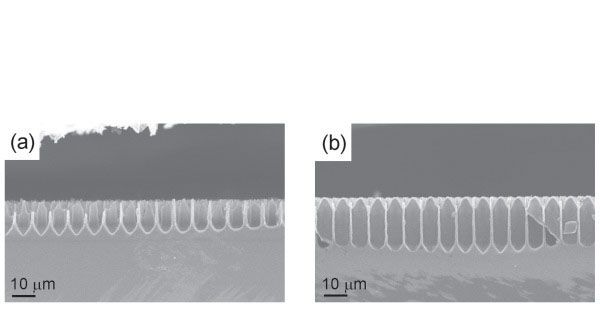
**Comparison of SEM micrographs of macropores formed in HF solutions with and without Et**_**2**_**O.** Cross-sectional views of porous layers anodized in a 10–20 Ωcm prepatterned p-type silicon at 14 mAcm^−2^ for 1 h in (**a**) HF:H_2_O:EtOH with a composition of 5:6:29 and (**b**) HF:H_2_O:EtOH:Et_2_O with a composition of 5:6:15:14 in volume.

When Et_2_O was added to HF solutions with alcohol, the stable macroporous array was obtained. Figure 4 shows cross-sectional views of porous layers anodized in ca. 8 wt.% HF solution with only EtOH or EtOH + Et_2_O. This effect was observed in HF solutions with all the alcohols. Even with MeOH solution, in which macroporous layer was hardly obtained only with alcohol, macropores were formed due to the presence of Et_2_O.

As the number of carbon in alcohol increases, stable macropore formation is achieved when silicon is anodized in low-concentration HF solution. Macropores formed with alcohol become stable against the surface dissolution, as the carbon chain becomes longer in alcohol. Though the dissolution of the top surface appears in ca. 8 wt.% HF solution with alcohol, this dissolution is not observed on the sample anodized in ca. 30 wt.% solution with alcohol. These results indicate that the top surface dissolves only in the low concentration of HF. Silicon can be dissolved via oxide when the low-concentration HF solution is used [10]. Also, some organic solvents can oxidize silicon [10]. The top surface may dissolve via oxide during formation of porous layer.

When Et_2_O is added to the HF solutions with alcohol, stable macroporous layers are obtained even with MeOH, though macropores are not formed in MeOH solution without Et_2_O. As discussed above, macropore growth becomes stable with the increasing number of carbon atoms in alcohol. In addition, Et_2_O also stabilizes the macropore formation. The polarity of alcohol becomes lower with the increasing number of carbon in alcohol. Et_2_O also has low polarity. A polarity of the solvent seems to play a decisive role for the macropore formation. The pore wall is hydrophobic because the wall surface is hydrogen-terminated. Then alcohol having a long carbon chain and Et_2_O are likely to be accommodated in the vicinity of pore walls, and the state leads to a low density of water and/or HF on the wall surface. When alcohol which has a long carbon chain or together with Et_2_O is used, the dissolution rate on the silicon surface becomes low because of the low density of HF in the vicinity and stable pore formation is achieved. Chao et al. reported that amphiphilic surfactants adsorbed on the surface of the electrode with their nonpolar tails attached on the hydrophobic surface; the adsorption may protect the sidewalls against dissolution [11]. On the other hand, with alcohol having a short carbon chain and without Et_2_O, relatively high concentration of HF can be expected in the vicinity compared with the low polar solvents. Therefore, a high polar solvent such as MeOH results in the nearly uniform distribution of HF on the surface, i.e., the concentration of HF seems to be not so different at the pore bottom and elsewhere. That is the reason why macropores cannot be obtained when those types of alcohol are used.

**Figure 5:**
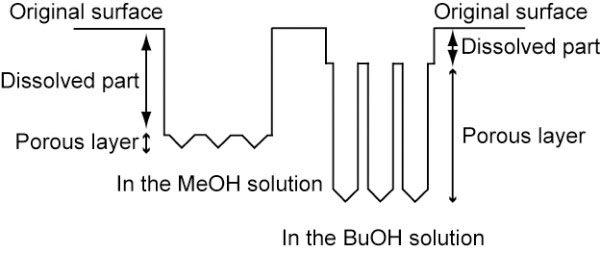
**A schematic of different dissolution modes.** A schematic cross-sectional view illustrating porous layers anodized in the MeOH and BuOH solutions.

In spite of the difference in the dissolution of the top surface depending on the type of alcohol, the dependence of the mass loss on the type of alcohol is hardly observed during anodization. The electrochemical valence, which is calculated from the value of mass loss of silicon, is not so different with each other, where the electric charge is kept constant. Silicon dissolution can occur in two ways. The first route is the divalent electrochemical dissolution, which results in porous layer formation, and the second one is electropolishing, which has a valence of four [12]. If the dissolution occurs through various routes depending on the samples, the valence indicates different values. In this study, the valence shows almost the same value in all cases. These results exhibit that the dissolution reaction in this study proceeds through the same way with all types of alcohol. Figure 5 summarizes the results obtained in this study schematically. The depths of dissolved part at the top surface and porous layer depend on the type of alcohol. Judging from the SEM observation, the total thickness of the dissolved part and the porous layer formed with MeOH is quite thin (ca. 23 μm), while it becomes thicker (ca. 33 μm) in BuOH. As mentioned above, the electric charge is the same, and the mass loss of silicon also shows almost a constant value in all cases. These results indicate that noticeable chemical dissolution does not occur. This is why silicon dissolves deeper in the BuOH solution, preventing it from the dissolution of pore walls, since keeping the pore walls can save additional charge to be used for the pore growth toward the depth direction.

The tetravalent dissolution sometimes occurs via oxide [10,12]. In this study, the measured valence is around 2.5. The value over two implies that tetravalent dissolution or electrochemical etching takes place partially. The preferential site should be the pore tip because the current collector is located at the back of the silicon substrate, and the pore tip has the small curvature. An oxide formation at the pore tip and its dissolution therefore leads the pore growth to a vertical direction. Besides, the dissolution along this direction can prevail over other directions, since water or HF tends to be provided for the oxide which is hydrophilic. As of now, we have not studied the details yet. This subject will be studied in the future.

## Conclusions

As the number of carbon in alcohol in a low concentration HF solution increases, the stable macropore formation is achieved. Macropores also become stable against the surface dissolution under the same condition. The dissolution of the top surface is caused by the presence of alcohol in the low HF concentration solution. When the solution containing Et_2_O is used, the dissolution of pore walls is suppressed. It is confirmed that the type of alcohol has a significant effect on the pore formation. Not only current density or concentration of HF solution but also the type of alcohol affects the pore structure. The property of organic solvents is crucially important for the porous layer formation.

## Competing interests

The authors declare that they have no competing interests.

## Authors’ contributions

TU performed most of the experiments and prepare the first draft of the manuscript. KF, TS, and YHO supervised this work. All the authors discussed the results and revised the manuscript. All authors read and approved the final manuscript.

## References

[B1] Theunissen MJJ Etch channel formation during anodic dissolution of n-type silicon in aqueous hydrofluoric acid J Electrochem Soc 1972 7 351 360 10.1149/1.2404201

[B2] Propst EK Kohl PA The electrochemical oxidation of silicon and formation of porous Si in acetonitrile J Electrochem Soc 1994 7 1006 1013 10.1149/1.2054832

[B3] Wehrspohn RB Chazalviel J-N Ozanam F Solomon I Electrochemistry and photoluminescence of porous amorphous silicon Thin Solid Films 1997 7 5 8 10.1016/S0040-6090(96)09362-5

[B4] Ponomarev EA Lévy-Clément C Macropore formation on p-type Si in fluoride containing organic electrolytes Electrochem Solid St 1998 7 42 45

[B5] Lehmann V Rönnebeck S The physics of macropore formation in low-doped p-type silicon J Electrochem Soc 1999 7 2968 2975 10.1149/1.1392037

[B6] Carstensen J Christophersen M Föll H Pore formation mechanisms for the Si-HF system Mater Sci Eng B 2000 7 23 28

[B7] Harraz FA Kamada K Kobayashi K Sakka T Ogata YH Random macropore formation in p-type silicon in HF-containing organic solutions—host matrix for metal deposition J Electrochem Soc 2005 7 C213 C220 10.1149/1.1864292

[B8] Song JH Sailor MJ Dimethyl sulfoxide as a mild oxidizing agent for porous silicon and its effect on photoluminescence Inorg Chem 1998 7 3355 3360 10.1021/ic971587u

[B9] Hamm D Sasano J Sakka T Ogata YH Silicon anodization in HF ethanoic solutions—competition between pore formation and homogeneous dissolution J Electrochem Soc 2002 7 C331 C337 10.1149/1.1473778

[B10] Christophersen M Carstensen J Voigt K Föll H Organic and aqueous electrolytes used for etching macro- and mesoporous silicon Phys Status Solidi A 2003 7 34 38 10.1002/pssa.200306464

[B11] Chao KJ Kao SC Yang CM Hseu MS Tsai TG Formation of high aspect ratio macropore array on p-type silicon Electrochem Solid St 2000 7 489 492

[B12] Föll H Leosner M Cojocaru A Carstensen J Macroporous semiconductors Materials 2010 7 3006 3076 10.3390/ma3053006

